# Fine-Scale Aerosol-Jet Printing of Luminescent Metal–Organic Framework Nanosheets

**DOI:** 10.1021/acsami.4c10713

**Published:** 2024-10-04

**Authors:** Dylan A. Sherman, Erik Landberg, Anjana Ramesh Peringath, Sohini Kar-Narayan, Jin-Chong Tan

**Affiliations:** † Multifunctional Materials & Composites (MMC) Laboratory, Department of Engineering Science, University of Oxford, Parks Road, Oxford OX1 3PJ, U.K.; ‡ Department of Materials Science & Metallurgy, University of Cambridge, 27 Charles Babbage Road, Cambridge CB3 0FS, U.K.

**Keywords:** metal−organic framework nanosheets, aerosol-jet printing, luminescent thin films, white-light-emitting diodes, micropatterning

## Abstract

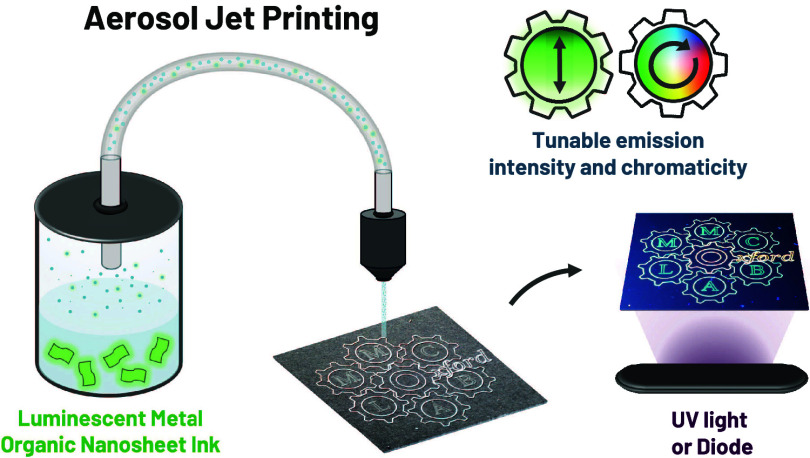

Fabrication of metal–organic framework (MOF) thin films is an ongoing challenge to achieve effective device integration. Inkjet printing has been employed to print various luminescent metal–organic framework (MOF) films. Luminescent metal–organic nanosheets (LMONs), nanometer-thin particles of MOF materials with comparatively large micrometer lateral dimensions, provide an ideal morphology that offers enhancements over analogous MOFs in luminescent properties such as intensity and photoluminescent quantum yield. The morphology is also better suited to the formation of thin films. This work harnesses the preferential features of LMONs to access the advanced technique of aerosol-jet printing (AJP) to print luminescent films with precise geometries and patterns across the micrometer and centimeter length scales. AJP of LMONs exhibiting red (R), green (G), and blue (B) emission were studied systematically to reveal the increase of luminescence upon additive layering printing until a threshold was reached limited by self-quenching. By combining different LMON emitters, emission chromaticity and intensity were shown to be tunable, including the combination of RGB emitters to fabricate white-light-emitting films. A white-light LMON film was printed onto a UV light emitting diode (LED), producing a working white-light-emitting diode. Printing with multiple distinct photoluminescent inks produced intricate multicolor patterns that dynamically responded to excitation wavelength, acting either as micrometer-scale LED-type cells or larger visual tags. Collectively, the work offers an advancement for MOF thin films by printing MON materials using AJP, offering a precise method for manufacturing a wide range of critical functional devices, from luminescent sensors to optoelectronics, and more broadly even the opportunity for printed circuitry with conductive MONs.

## Introduction

Metal–organic framework (MOF) materials are evolving with enhanced properties ideal for microscale sensors, electronics, and organic light-emitting diodes (OLEDs).^1−3^ A lack of fabrication techniques with control at the microscale and smaller, however, is a significant drawback to integrating MOFs into devices for these applications.^4^ White-light-emitting (WLE) MOFs for organic LEDs (OLEDs), for example, are typically fine powders with intrinsic nonthermoplastic properties, brittleness, and insolubility. A common type of luminescent MOF design involves trapping luminescent guest molecules in the cavities of the MOF framework (guest@MOF).^5^ The frameworks typically enhance guest stability and offer tunability for the luminescent properties of the guest. These particular WLE materials have the added difficulty of avoiding guest leaching and dilution due to solvent infiltration during material processing.^6^ Thus, processing MOF nanocrystals into specific films that are structurally robust and exhibit operational flexibility is a highly desired area of exploration.^4,7^

Most WLE MOF diode-based devices utilize downward conversion from electric power to a blue LED or UV chip, which is absorbed by the MOF composite (yellow- or white-emitting, respectively) to generate WLE.^4^ A few also integrate WLE MOF materials into electroluminescent devices.^8^ Both methods typically require thin films of MOF composites, and their performance depends on film thickness and surface roughness, which influences charge transport.^8^ Most successful WLE MOF works produce a rough and uneven deposit of powder for proof-of-concept MOF emission on UV chips.^9−14^ Gong et al. instead dip-coated a 5 mm blue LED blub with a thin film of [Zn_6_(btc)_4_(tppe)_2_(DMA)_2_], a WLE LMOF prepared via suspension in ethyl acetate followed by sonication.^15^ Our lab has developed methods to circumnavigate these challenges by preparing a guest@MOF yellow emitter three-dimensional (3D)-printed in a blue-emitting polymer resin and electrospinning high photoluminescence quantum yield (PLQY) MOF@fiber composites.^16−18^

A more recent method for WLE guest@MOF fabrication that offers improved control and precision is patterning by printing. MOF framework printing is dominated by direct ink-writing,^19−21^ with a small selection of luminescent MOF materials successfully inkjet printed for functions including anticounterfeiting, watermarks, and sensors.^22^ The process is additive so layers can be added sequentially, useful for applications such as security and climate or counterfeit tags on packaging, sensor bioarrays, or OLED display pixels. Considerations such as the ink viscosity, surface tension, and interaction between ink and target substrate are critical variables for successful printing.^23^ Success is often impeded by the particle size of the crystalline powders and material instability, which can lead to agglomeration, during printing in solvent.^24−26^

Aerosol-jet-printing (AJP) is a fast-growing alternative manufacturing system that offers increased resolution, flexibility, and printing speed at the micron to nanoscale.^27^ The noncontact technique involves the aerosolization of a functional ink that is deposited onto a substrate with resolutions up to 10 μm (typically between 30 and 50 μm).^27−29^ Controlled digitally, fine microscale patterns can be achieved in 3D using additive manufacturing, with works achieving lattices and micropillar arrays.^30^ While initially used for printing circuitry, the advent of new functional inks has led to printing more complex electronics, displays, and sensors for health and environmental applications.^31,32^ Only a few works have reported the printing of luminescent materials. These include printed lanthanide-doped upconversion nanoparticles inks luminescent under NIR excitation;^33^ electroluminescent inorganic OLED lines of red, green, and blue with 30 μm width for 140 ppi resolution screens;^34^ europium-doped yttrium oxide nanospheres for red luminescent patterning;^35^ one work on printing LED modules;^36^ and printing of UV luminescent quantum dots (5, 32, and 23% QY for blue, green, and red, respectively).^37,38^ Studies have yet to systematically examine how luminescence as a property can be modified as a function of printing parameters.

MOFs are ideal candidates for tunable functional inks, but AJP is limited by particle size being 50 nm or less to allow for ultrasonic atomization.^39^ Particle aggregation also impacts the uniformity of particle size distribution, inhibiting effective atomization or further on printing itself. Only one report, to our knowledge, uses AJP related to a MOF. The work by Kravchenko et al. relies on reagent inks to overcome particle printing limitations so that an ultraporous calcium squarate framework forms in situ after printing.^40^ Metal–organic nanosheets (MONs), MOF particles with a few nanometer-thin thickness but micrometer-wide planar dimensions, offer a morphology more suited to micro fabrication. Recent works also show how MONs such as ZIF-7-III (two-dimensional (2D) sheets) can be functionalized with guest incorporation to achieve luminescent MON (LMON) materials with particles of 2–5 nm thickness.^41^ Emission colors ranged across the visible spectrum and included optimal WLE.^42^ The zinc-based ZIF-7-III 2D nanosheets offer enhancements such as increased intensity and quantum yield over analogous guest@ZIF-7 3D materials due to increased surface area, optical transparency, and improved interguest cooperative behaviors due to interlayer packing configurations.^41,42^ The fabrication of LMONs into thin films is a yet-to-be-explored opportunity.

This work utilizes organic dyes@ZIF-7-III LMON materials (dyes@Z7-NS) to demonstrate the first direct aerosol-jet printing of a MOF material to produce micrometer-scale control of thin film luminescent patterning. The potential for 3D LMON pillaring for directing emission is also illustrated. By systematically studying the emission properties of micropatterns with fluorescence microscopy, we sought to establish property relationships that examined the effect of printing strategies on the emission properties of printed LMONs.

## Methods

All of the reagents, solvents, and chromophore guests were commercially acquired from Sigma-Aldrich, Fisher Scientific, and Alfa Aesar, and used as received.

### Material Synthesis (Dye@Z7-NS)

Material synthesis (dye@Z7-NS) as adapted from Sherman et al.^41^ Zn(NO_3_)_2_ (59.5 mg, 0.2 mmol) was dissolved in methanol (MeOH) (2 mL). The solution was added to NaCl powder (20 g) under vigorous magnetic stirring. Benzimidazole (bIm) (283.5 mg, 2.4 mmol) was dissolved in MeOH (2 mL). The chromophore guests (7-methoxycoumarin (MC), fluorescein (F), and/or rhodamine B (RB)) were solubilized with MeOH (2 mL) via sonication and mixed with the bIm solution. After 20 min, the solution was added dropwise to Zn(NO_3_)_2_@NaCl followed by vigorous stirring for 12 h. Deionized H_2_O (250 mL) was added to the flask and heated to 50 °C while stirring. The mixture was siphoned into 50 mL centrifuge tubes and centrifuged at 10,000 rpm for 20 min, followed by further washing and centrifuging cycles (3 × 45 mL of H_2_O and 5 × 40 mL of MeOH). The final product was dried for 1 h at 90 °C (≈60–70% yield).

### Ink

Ink suited for aerosol-jet printing was created by dispersing guest@Z7-NS in toluene to a concentration of 40 mg/mL followed by ultrasonication and vortexing. Optimal ultrasonication and vortexing times were found to be 10 min and 30 s, respectively.

### Aerosol-Jet Printing

Aerosol-jet printing was performed using an Optomec AJ200 fitted with a UA Max ultrasonic atomizer to atomize the ink and deposit it on various substrates, primarily poly(ethylene terephthalate) (PET) film and glass slides. Sheath flow rate (sccm)/atomizer flow rate (sccm) determines the focus ratio for the deposition. The following AJ200 settings were generally used for deposition; A start-up focus ratio of approximately 1 until material deposition was achieved followed by a printing focus ratio of 5:9 to achieve a more focused deposition. Printing speed was 0.5 mm/s on glass and 0.2 mm/s on PET (0.35 mm thickness). A 150 μm tip nozzle was used with a working distance of 1 mm. The ultrasonic atomizer current was set between 0.5 and 0.6 A with the ultrasonic atomizer cooling water temperature at 14 °C. The substrate area (platen) was set to 70 °C. For pillar formation, a focus ratio of 0.375:1 was used to increase material deposition and reduce the risk of pillar breakage due to high gas flow.

### Fluorescence Microscopy

Fluorescence microscopy images were collected using a Zeiss LSM780 with a 10× confocal lens. Lambda scanning (at a resolution of either 9 or 3 nm) was used to image fluorescence with laser lines of either 405 nm (diode source), 488 nm (argon multiline 25 mW), or 543 nm (HeNe 1 mW). Data was analyzed using Zeiss ZEN 3.9.

### Scanning Electron Microscopy (SEM)

Scanning electron microscopy was performed using a Hitachi SEM (TM3030 Plus 0865). Field-emission SEM (FE-SEM) was obtained at 10 keV under high vacuum using a Tescan Lyra 3 (Tescan, Czech Republic) with secondary and backscattered electron imaging (SEI and BSE, respectively) under 10–15 keV.

### White-Light Emission Spectra

White-light emission spectra were collected using an Ocean Insight spectrometer. The output signal was carried by optical fiber, with one exposed end of the fiber 3 cm above the emitting diode, to the Ocean Insight FLAME-T-UV–vis-ES miniature spectrometer (200–850 nm). The spectrometer was connected to a computer running OceanView software for data logging and analysis. The diode was placed in a 3D-printed enclosed cylinder to prevent ambient light from interfering with measured signal.

### Surface Roughness and Micropillar Dimensions

Surface roughness and micropillar dimensions were obtained using an InfiniteFocus Alicona optical profilometer. Images of the AJP prints were collected using a 20× objective lens at a vertical resolution of 50 nm and a lateral resolution of 3.5 μm. Data analysis, including height profiling and surface roughness, was computed using the Alicona IF Measure Suite. Surface roughness conformed with ISO 4287, using an area of width 30 μm and length 200 μm (26 profiles).

## Results and Discussion

### Ink Development and Characterization

To create an LMON-based ink, the LMON particles were dissolved in a suitable solvent. Determining the solvent and optimal ink parameters is a sensitive process,^28^ which required tuning of solvent and particle wt % before then modifying instrument parameters. While common solvents for AJP such as methanol, ethanol, and isopropanol were tested in various quantities and mixed ratios, toluene was found to be the ideal LMON ink solvent. One reason for this is its aprotic nonpolar character, limiting interaction with the Z7-NS dispersion, thereby discouraging aggregation. Lower volatility compared to that of methanol and isopropanol also meant that the ink remained more stable during printing, maintaining a good dispersion of LMON particles. To achieve proper deposition, the substrate platform was heated to 70 °C to assist in evaporation. Toluene viscosity at room temperature (0.68 cP at 20 °C) is below the optimal AJP viscosity window of 1–5 cP,^39^ however cooling the ink to 14 °C achieved a viscosity appropriate for deposition. To determine the appropriate wt % of LMON in toluene, a range of values were tested for each dye (10, 20, 30, 40, and 50 mg/mL) across multiple layering (1, 2, 4, 8, 16, 32). Data showed beyond 40 mg/mL that the concentration of LMON particles was too high to effectively print. Below 40 mg/mL, the prints were undefined and inconsistent (see Figure S1). A concentration of 40 mg/mL was therefore selected for this study, with inks prepared in batches of 1.5 mL. PET film of 0.35 mm thickness was used as the substrate over glass slides, as the PET film was found to exhibit improved particle adhesion. Due to its thinness, flatness, and transparency, it is also ideal to place as a film over diodes or similar optical devices. The film was treated with EtOH as per previous studies to improve contact angle and increase particle adhesion further.^43^

Extensive trials were undertaken to optimize the focus ratio to produce prints with the highest resolution. Initial trials using low focus ratios resulted in diffuse and uneven printing (Figure S2). Ultimately a two-step focus ratio method was used to minimize deposition variability (see the Methods section), with precise parameters requiring minor adjustments given the inherent variability of the Optomec AJ200. This involved initiating the system with a low focus ratio (≈1) to initially create a flow of LMON particles. Before printing, the focus ratio was increased to 6–9, producing a consistent focused print with the least overspray possible. Depositions at high focus ratios that lasted over 1.5 h could be achieved without interference. For prints over 2 h (at a rate of 0.2 mm/s), incremental deposits of LMON formed on the walls of the ink vial and in the transfer tubing. This required redispersing the material in solvent from the walls and ensuring the set ink concentration remained.

**Figure 1:**
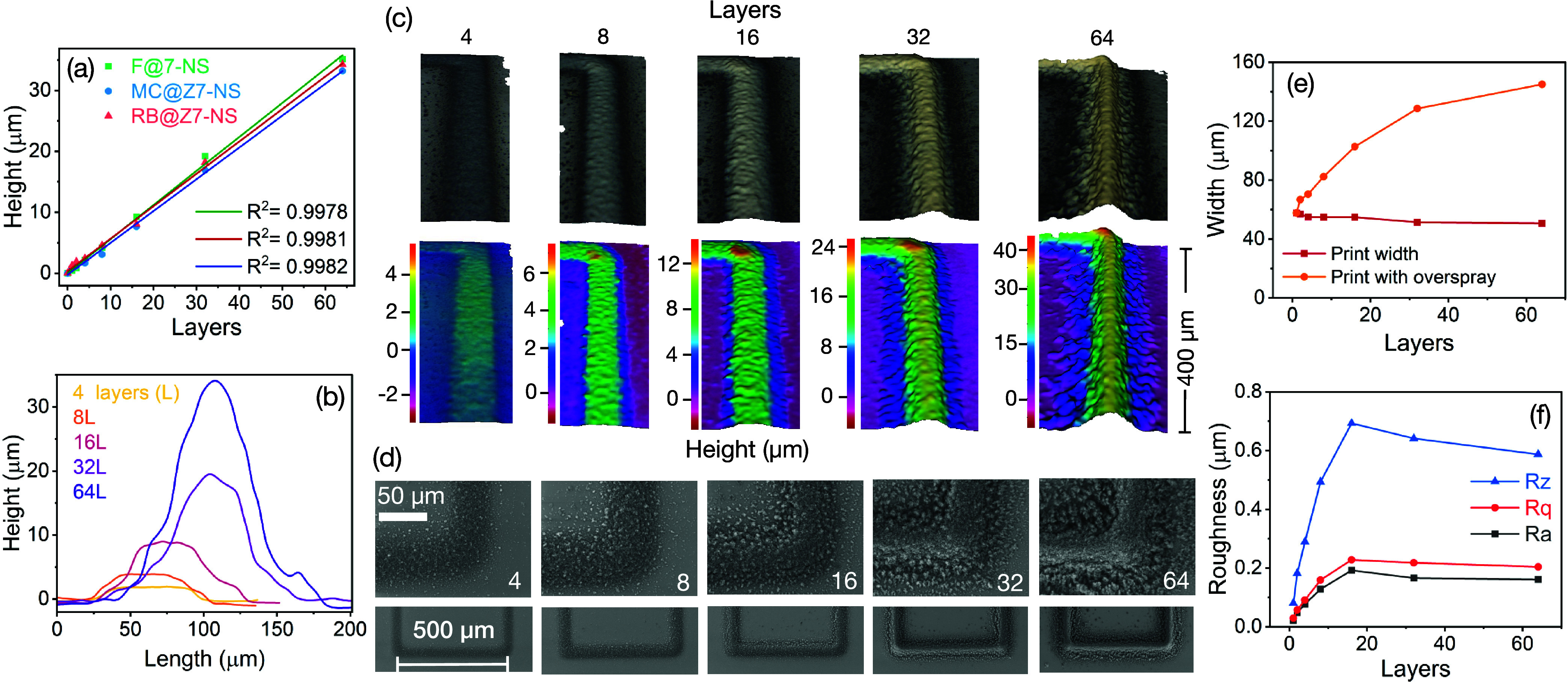
Characterization of aerosol-jet-printed luminescent metal–organic nanosheets (LMON). (a) Relative maximum heights of different LMON inks printed, showing linear relationships between the number of layers printed and printed pattern height. (b) Height profiles perpendicular to the printed line for F@Z7-NS prints of various layers. (c) Surface profiles of a single edge of F@Z7-NS square perimeter prints (500 × 500 μm^2^) with various layers, presented as optical images (top) and height heat maps (bottom). (d) SEM of corresponding prints at various layers, showing print corners (top) and edge (bottom). (e) F@Z7-NS print widths (defined as width at 50% height, and complete width at base of height profile to include overspray), based on an average along one square edge of each print. (f) F@Z7-NS print surface roughness measured as *R*_z_ (mean distance between highest and lowest points on surface), *R*_a_ (mean), and *R*_q_ (root-mean-square) at varying layering.

To investigate the topography and printing quality of the optimized LMON prints, a series of printed square perimeters with edge length of 500 μm and varying layers (1, 2, 4, 8, 16, 32, 64) were prepared for three key LMON inks: the green emitter fluorescein@Z7-NS (F@Z7-NS), the blue emitter 7-methoxycoumarin@Z7-NS (MC@Z7-NS), and the red emitter rhodamine B@-7-NS (RB@Z7-NS). The prints were characterized using optical surface profiling and scanning electron microscopy (SEM). The height of the prints (averaged along one square perimeter side of each print) was found to increase linearly with layers, with a maximum thickness of 32–34 μm (Figure 1a). This implies that each layer was of a similar consistency and height for all inks (0.53 ± 0.01 μm), suggestive of a reliable printing process; all layered prints of each LMON ink were printed in one session with no ink adjustment or intervention.

Investigating the height profiles of F@Z7-NS more closely, as an example, revealed a sequential transition from more rectangular surfaces with minimal overspray to a defined peak-shaped surface but with increased overspray (Figure 1b). Data show consistency along the line prints of each layer quantity (Figure 1c,d). These visual features were quantified by measuring printed line thickness at 50% of the maximum height to represent the line, compared to overspray at the lowest height profile width (Figure 1e). Data show that while the print width decreases from 57 to 50 μm with increasing layering, overspray significantly increases from effectively 0 to 95 μm (total overspray adding both edges of the print). Surface roughness of the samples revealed an increase in roughness up to prints of 16 layers, followed by a slower decrease thereafter. This characterization suggests that depending on the resolution and shape of line desired, layering can be used to produce a flatter more even surface, or a more defined narrower surface, but with increased overspray. Of note, as these prints are intended for luminescent studies, the extent of overspray and print quality observed under ambient conditions is less significant than how these prints appear under UV and the effect any overspray and print shape will have on the observed luminescence. Indeed, increasing surface roughness exposes more surface area, which theoretically can increase the intensity of fluorescence emission. Notably, while not as precise as traditional AJP inks such as silver,^44^ the consistency, control, and quality of these LMON AJP prints are comparable to more recently emerging sensitive organic/hybrid AJP inks in the literature (e.g., bioorganic inks and europium oxide phosphors).^45,46^ They show significant improvement over the precision and repeatability obtained with inkjet printing LMON and LMOF materials.^42,43,47,48^

### Controlling Luminescence through LMON AJP

What follows is a series of strategies that are systematically employed to examine how certain patterning and additive printing techniques can be used to tune the luminescence properties of the LMON ink. The work is compared to the previously established photophysical properties of the LMON materials in the literature.^41,42^

#### Strategy 1: Layering Homogeneous LMONs

**Figure 2:**
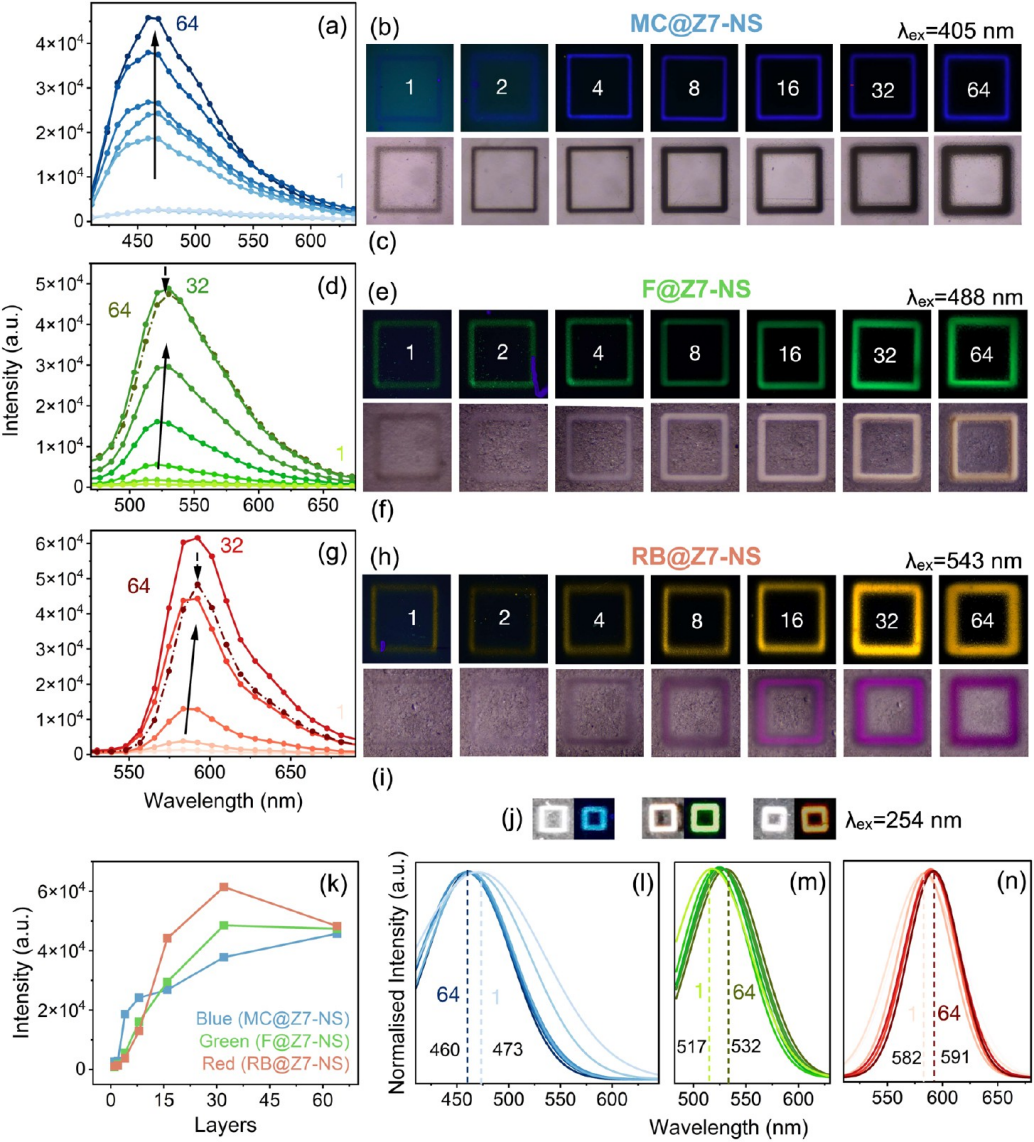
Fluorescence microscopy of AJP luminescent guest@Z7-NS square perimeter prints of side length 500 μm. Any dust or contamination on the substrate appears as bright purple marks. (a) Lambda scanning emission spectra from fluorescence microscopy for AJP MC@Z7-NS excited at 405 nm with increasing layering from 1 to 64 indicated by darkening of data points and lines. (b) Corresponding microscopy imaging of MC@Z7-NS prints excited at 405 nm, with numbers indicating layers in each image and color computed by Zeiss software, representing the intensity and chromaticity derived from the emission spectra. (c) Optical microscopy images of corresponding MC@Z7-NS prints. (d–f) Data as in (a)–(c) but for AJP of F@Z7-NS excited at 488 nm. (g–i) Data as in (a)–(c) but for AJP of RB@Z7-NS excited at 543 nm. (j) 32-Layer printed samples under ambient (left) and 254 nm UV light (right) for MC@Z7-NS, F@Z7-NS, and RB@Z7-NS (left to right). (k) Intensity of emission maximum for each print, relative to the 64-layering intensity. (l–n) Normalized and Gaussian fit spectral curves indicating any peak shift from 1- to 64-layer prints for MC@Z7-NS (left), F@Z7-NS (middle), and RB@Z7-NS (right).

We first examined the luminescent properties of the additive layering of the same LMON material using the square perimeter prints of side length 500 μm and the selection of 1, 2, 4, 8, 16, 32, and 64 layers. LMON inks included the green-emitting F@Z7-NS, red-emitting RB@Z7-NS, and blue-emitting MC@Z7-NS (Figure 2).

Fluorescence microscopy was employed to study the prints (Figure 2a–h), with lambda scans producing emission spectra for each print (Figure 2a,d,g), along with corresponding imaging of each print under laser excitation (Figure 2b,e,h) and ambient conditions (Figure 2c,f,i). Due to discrete laser wavelengths available, 405 nm was the highest energy excitation laser accessible to excite MC@Z7-NS while 488 nm was used for F@Z7-NS and 543 nm for RB@Z7-NS. These excitation wavelengths align well with the excitation ranges of each sample (Figure S3). The fluorescence imaging of each print was colored using Zeiss Zen software, calculated based on the emission profile of each print to simulate the color and intensity. To confirm these simulations against observed emission color, prints of 32 layers of each LMON were placed under a UV lamp (4 W, 254 nm) and photographed with zoom (Figure 2j).

Spectra (Figure 2a,d,g) confirm that all three LMONs emit consistent with the previously reported emission spectra of the LMON in powder form.^41^ Line scans across the microscopy imaging confirm the homogeneity of the emission intensity along the patterning (Figure S4), with only minor intensity increases at the corners to be expected from the printing nozzle changing direction resulting in increased print thickness (seen also in Figure 2c). All prints (Figure 2b,e,h) show clear and defined luminescent patterns, with improved definition resulting from increased layering. While overspray was found to increase noticeably with layering, along with a shift from rectangular to peak-shaped topography, these adjustments appear to have minimal impact on the definition of the luminescence pattern observed in fluorescence imaging with little edge ghosting or shadow effects observed (see also photographs in Figure 2j). This is likely due to the relative minimal emission intensity contribution from overspray areas due to lower comparable thickness. Analysis of emission intensity perpendicular to F@Z7-NS line prints of varying layers (Figure S5) reveals single broad emission profiles (compared to profiles with multiple peaks and local maxima), supporting the observed defined visual appearance in imaging. These broad emission intensity profiles correspond to the overall line width of the prints, including overspray.

The PET substrate is emissive, with excitation λ_max_ = 340 nm and a corresponding broadband emission band from 300 to 600 nm with λ_max_ = 385 nm (Figure S6). In operating conditions, a 285 nm UV energy source was employed to avoid any noticeable contribution from the PET. The highest energy laser accessible for microscopy, however, was 405 nm and was used in this study to best represent operating conditions. Hence, the chromaticity of emission observed for each sample is blue-shifted by the emission contribution from the PET substrate, the intensity of which is dependent on the laser wavelength. For reference, images of each material with 32 layers under a 254 nm UV lamp (4 W, 5 cm from sample) taken with a digital camera and zoomed show each material’s “true” operational color (Figure 1g).

Emission intensity from fluorescence imaging was normalized across different LMONs to the maximum layered sample of each LMON, achieved by adjusting the power of each excitation laser. This allowed for comparison not only between the layering of each LMON but also between different LMONs (Figure 2k). The maximum emission intensity for MC@Z7-NS increased mostly linearly with layer increase, apart from a sharp increase from 2 to 4 layers (Figure 2a). This is attributable to MC@Z7-NS being the weakest emitter, resulting in the patterns with the lowest material quantity exhibiting low intensity below the threshold of sensitivity of the detectors used. In contrast, the intensity of F@Z7-NS (Figure 2d) and RB@Z7-NS (Figure 2g), being stronger emitters, increases more linearly until 32 layers at which point intensity decreases for 64 layers. Given the linear increase in thickness with added layers (Figure 1a), this indicates there is a thickness threshold after which self-absorption by the bulk pattern dominates in competition with the intensity increase resulting from adding more LMON.

As a resolution of 9 nm was used to collect spectra, Gaussian peak fitting was employed to compare any peak maxima when analyzing potential energy shifts (Figure 2l–n). Fits reveal MC@Z7-NS emission bands narrow with increasing layering (a product of low detection), while F@Z7-NS and RB@Z7-NS present consistent peak shape and a red shift of 15 ± 9 and 9 ± 9 nm, respectively. Previous reports of these materials identified increasing aggregation to be the cause of red shift as guest loading increased.^41^ As thickness increases with layering, there is increased particle content along the *z* axis, which may interfere with emission. This may create favorable conditions for increased interparticle guest interactions and further self-absorption, leading to enhanced aggregation effects seen in emission.

#### Strategy 2: Layering Mixed LMONs for Chromaticity Control

**Figure 3:**
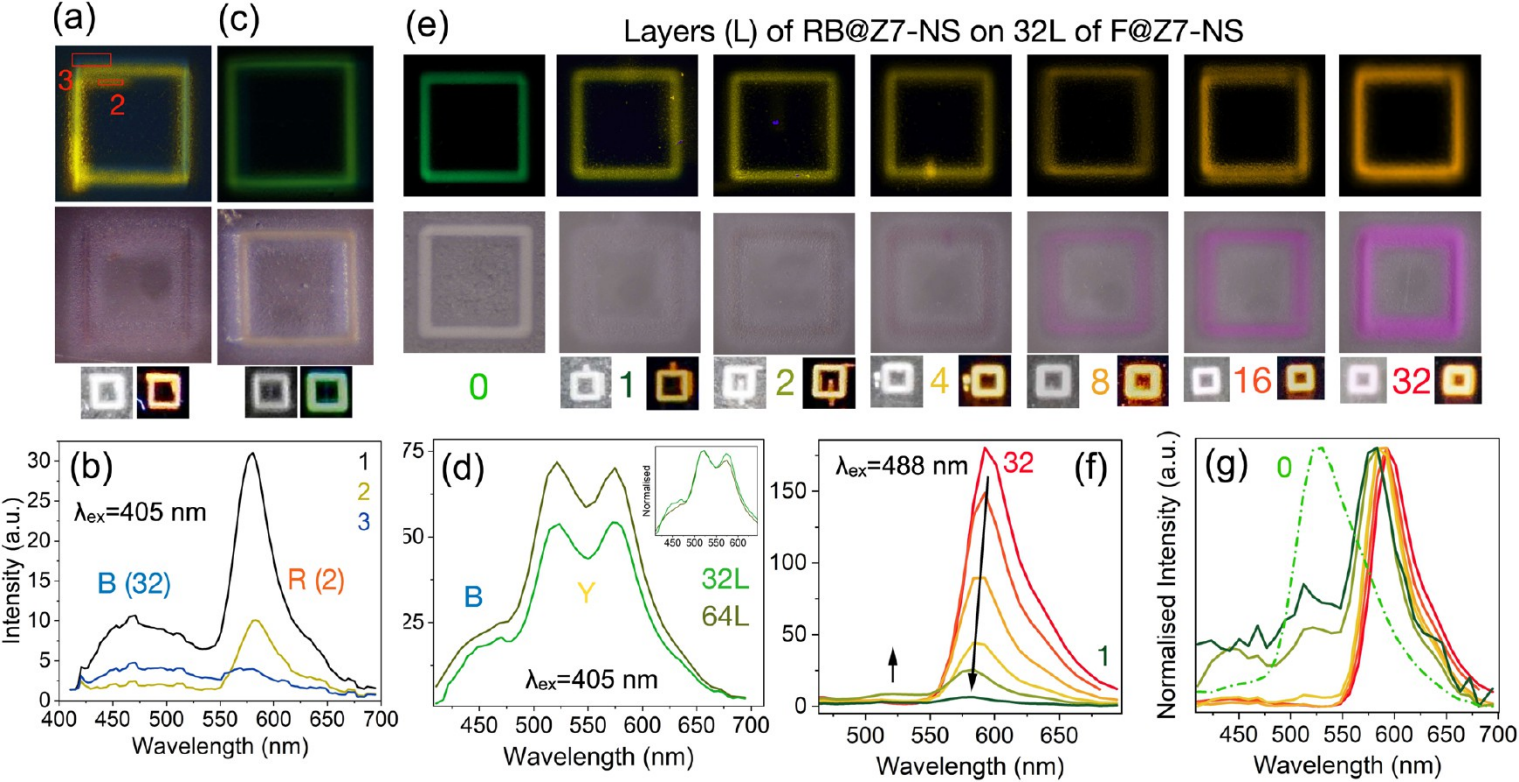
Square perimeter prints (500 × 500 μm^2^ each) with layering of different MONs. (a) Microscopy of AJP of MC@Z7-NS (32 layers) + RB@Z7-NS (2 layers) excited at 405 nm (above) and under ambient conditions (middle), with the sample viewed using a digital camera in the same conditions (bottom); (b) emission spectra from pattern imaged in (a). (c) MC@Z7-NS with F + RB@Z7-NS print (in the same layout as (a)), with corresponding emission spectra and normalized emission spectra (inset) (d). (e) F@Z7-NS 32-layer prints with a series of increasing RB@Z7-NS layers (in the same layout as (a)). Numbers indicate the number of RB@Z7-NS layers. (f) Corresponding emission spectra and normalized spectra (g) of (e), including the initial 32-layer F@Z7-NS spectra as a broken green line. Colors indicate the number of RB@Z7-NS layers per the coloring of numbers in (e).

Second, we investigated the additive patterning of different LMONs to mix emission colors. Blue (32 layers, MC@Z7-NS)- and red (2 layers, RB@Z7-NS)-emitting LMONs were combined to produce a yellow emitter (Figure 3a). Emission spectra (Figure 3b, 3 nm resolution, λ_ex_ = 405 nm) reveal regions where printing did not overlap and either blue emission (λ_max_ = 475 nm) or orange-red emission dominates (λ_max_ = 590 nm). Spectra averaged across the entire print exhibit a blue/red emission band of 1:3, highlighting the intensity of RB@Z7-NS as an emitter compared with MC@Z7-NS. In contrast, combining MC@Z7-NS with RB + F@Z7-NS (yellow), 32 layers each, produced a bright-green-emitting pattern (Figure 3c,d). The same was achieved when the solution was scaled up to 64 layers each (Figures 3d and S7). The emission bands of each material MC@Z7-NS:RB + F@Z7-NS were measured in a 1:3.3 ratio to achieve emission mixing (Figure 3d, 3 nm resolution, λ_ex_ = 488 nm). Notably, we also inverted the samples and undertook lambda scanning of the samples (i.e., exciting the lower blue-emitting MC@Z7-NS printed layers first), to confirm that the spectral data collected matched the reported upright data and the sample appeared consistent.

A systematic study was further completed by layering RB@Z7-NS sequentially on 32 layers of green-emitting F@Z7-NS (Figure 3e–g). Spectra (Figure 3f,g, 9 nm resolution) show that from one layer, the RB@Z7-NS emission band dominates, and by four layers, is effectively the only contributor to the overall material emission. Indeed, as the F@Z7-NS emission band overlaps with the RB@Z7-NS excitation band (Figure S8), it is likely that the emission from F@Z7-NS printed MONs is, to some extent, being reabsorbed and emitted by RB@Z7-NS. Fluorescence imaging of the patterns shows a progressive shift from green to yellow, then to orange and dark orange/red. Chromaticity stabilizes at around eight layers, with the printing of further RB@Z7-NS layers only increasing emission intensity. SEM imaging of 1-layer vs 2-layer RB@Z7-NS on 32-layer F@Z7-NS (Figure S9) reveals the distinctive LMONs, with a well-dispersed coating of RB@Z7-NS over the base F@Z7-NS layers.

#### Strategy 3: Mixed LMON Multipatterning

**Figure 4:**
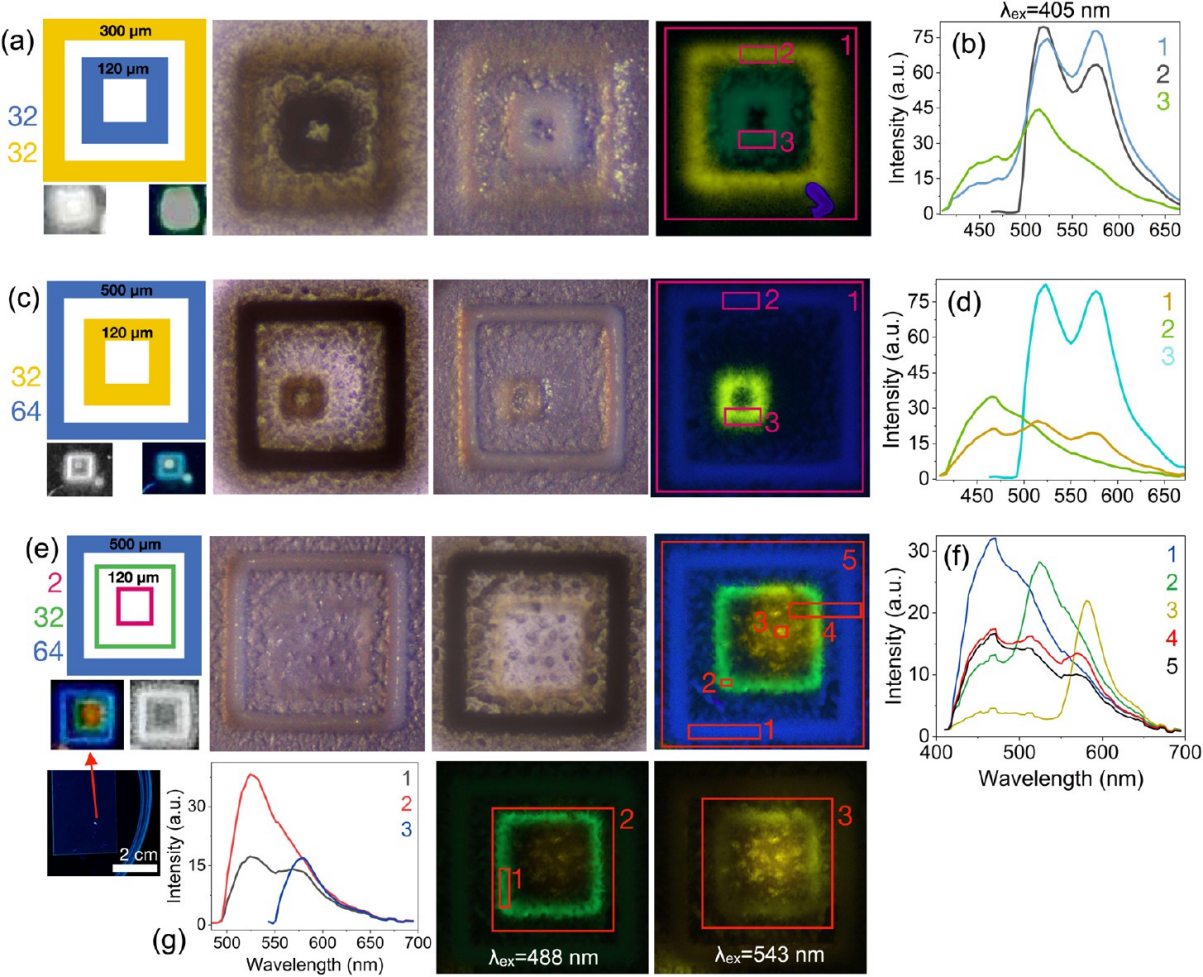
(a–d) MC@Z7-NS with F + RB@Z7-NS LMONs printed in various patterns of concentric squares (layers and dimensions indicated on the leftmost diagrams). Middle: optical microscopy images of prints under ambient conditions with uplighting (left) and downlighting (right). Right: microscopy images of prints excited at 405 nm. Bottom left images in (a) and (c) are taken with a digital camera to mimic observation by the eye under ambient (left) and UV light (right). (b, d) Emission spectra corresponding to highlighted squares in the rightmost image of (a) and (c), respectively. (e) MC@Z7-NS + F@Z7-NS + RB@Z7-NS multi-LMON print. Left: print conditions (leftmost diagram) with digital camera images below under UV (left) and ambient conditions (right) both zoomed and at eye level (lowest image). Middle: microscopy images of the print in (e) under optical ambient lighting (middle), and 405 nm excitation (right). (f) Emission spectra of print excited under 405 nm laser, with numbers corresponding to highlighted square sections of the print in (e). (g) Prints in (e) and (f) observed under 488 and 543 nm excitation showing microscopy images (right) and corresponding emission spectra (left). The spectral resolution is 3 nm.

Our third strategy involved the patterning of different materials at micrometer and larger centimeter scales. Blue- and yellow-emitting Z7-NS inks were printed as concentric square perimeters at two different scales. The smaller pattern (300 × 300 and 120 × 120 μm^2^) (Figure 4a) appeared green when photographed under UV, with lambda scanning revealing the dominant yellow dye contribution to emission (Figure 4b). In contrast, when the squares were more distinct (500 × 500 and 120 × 120 μm^2^) (Figure 4c,d), a blue square with yellow dot was visible when photographed and is differentiable by the eye (the average human eye resolution is 200 μm).^49^ Emission spectra (Figure 4d) confirm an equal balance of emission from blue and yellow emitters across the entire print, with distinct emission profiles of blue or yellow light at each respective square print. A similar print was achieved by combining F@Z7-NS (32 layers, 120 × 120 μm^2^) with RB@Z7-NS (1 or 2 layers 300 × 300 μm^2^) to produce orange-red bordered green emission dot (Figure S10).

Toward developing LED systems, we explored the combination of red-green-blue-emitting dyes in concentric squares. A test with ordering from outer-inner squares of red-green-blue confirmed the intensity of the red dye overpowered any delineation in the separate prints (Figure S11). In contrast, printing with blue-green-red order (64-layer MC@Z7-NS, 32-layer F@Z7-NS, and 2-layer RB@Z7-NS) produced distinctive concentric square patterning of the key color components required for a white-light-emitting LED, when photographed and (Figure 4e). By eye, the print appeared a pale blue-white color (Figure 4e). Reducing the layering by 50% (from 64:32:2 to 32:16:1 for blue/green/red) produced even more defined pattern, apart from the single layer of red being too diffuse (Figure S12). Emission spectra from microscopy with 408, 488, and 543 nm laser excitations (Figure 4f) showed the distinctive emissions of each dye pattern. Their dynamic nature is also demonstrated, such that certain components can be turned on/off depending on the selected excitation wavelength range. These patterns are highly promising for enabling the future fabrication of miniaturized LEDs (100–200 μm). There is a potential to even print Micro LEDs (<100 μm),^50^ if conditions and inks are optimized, given that a 40–50 μm line width was achieved.

**Figure 5:**
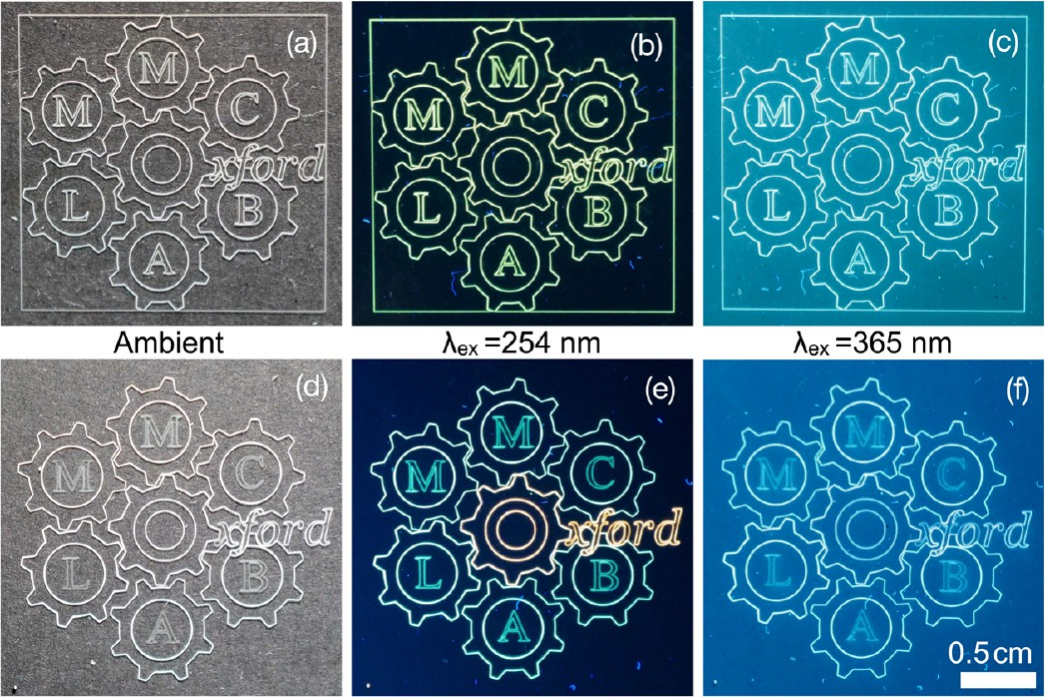
AJP print using yellow-emitting F + RB@Z7-NS LMON ink under ambient conditions (a), 254 nm UV light (b), and 365 nm light (c). AJP printed the logo using multiple LMONs of different emitters under ambient conditions (d), 254 nm UV light (e), and 365 nm light (f).

In contrast, we also applied the inks on a larger scale, first to produce green filled red square emitting patterns (Figure S13). The prints show a clear separation between the green-emitting filler and red-emitting border. More usefully, we printed a logo pattern at 2 cm × 2 cm scale using a single yellow dye and then a multicolored dye (green, yellow, orange/red) (Figure 5). The printing exhibits high definition of all pattern components, including text lines and complex cog intersections, while the multidye process does not cause any smudging. On the contrary, the central cog is a precise overlap of yellow ink with one layer of red ink, producing a lighter orange emission than the remaining orange/red text. Usefully, if the logo is exposed to UV light within the absorption range of the PET substrate, the colors are muted to display a homogeneous white-light-emitting logo (Figure 5c,f). Hence, it is possible to discretely encode information (e.g., distinct dye coloring) only viewable under a targeted subset of desired wavelengths.

#### Strategy 4: Pillaring for 3D Directional Emission Control

**Figure 6:**
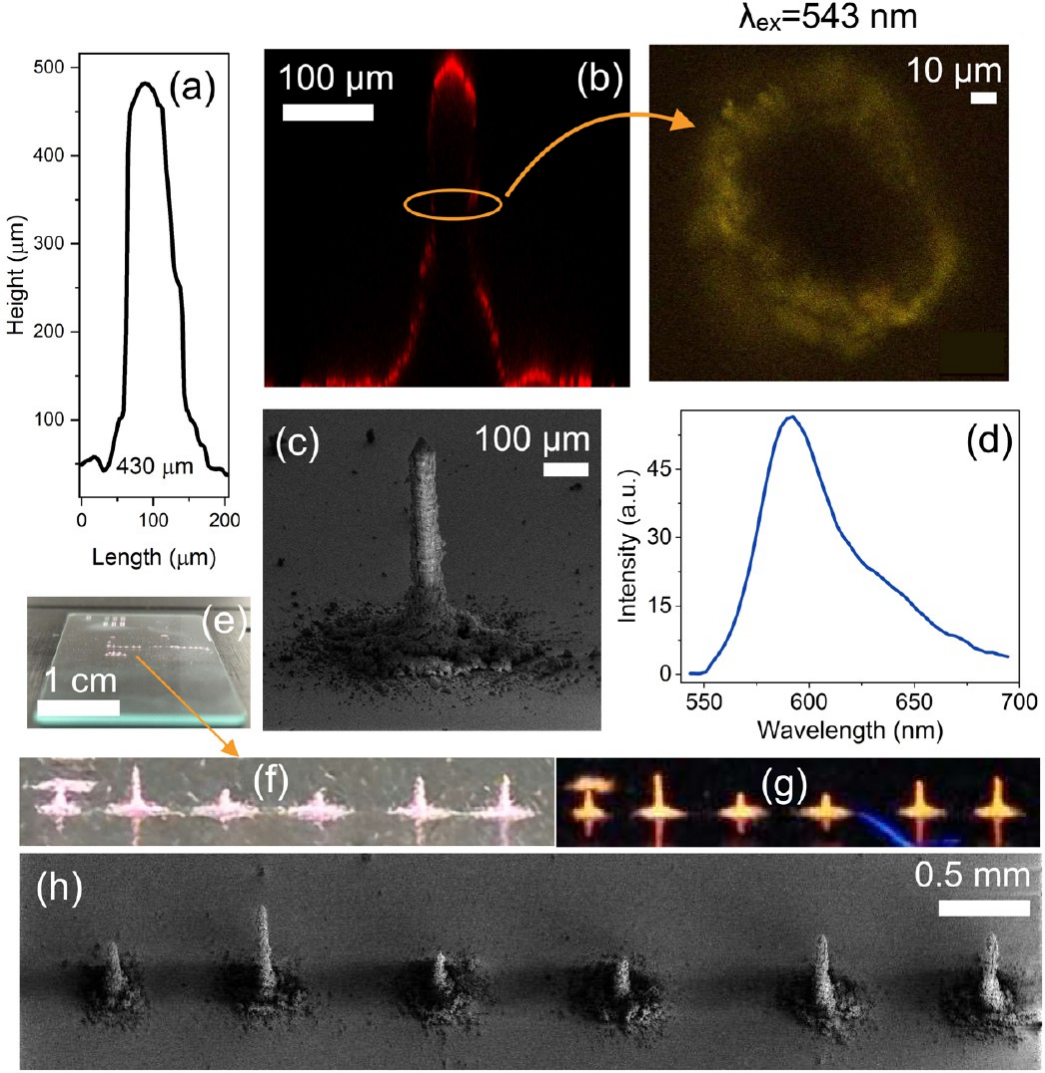
(a) Cross-sectional profile of the RB@Z7-NS printed micropillar. (b) Z-stack cross-sectional fluorescence microscopy image of the RB@Z7-NS micropillar excited with a 543 nm laser, including a horizontal slice through the pillar (right). (c) FE-SEM of RB@Z7-NS pillar. (d) Emission spectra from microscopy of RB@Z7-NS pillar. (e–g) RB@Z7-NS of various heights, under ambient (e, f) and UV (g) conditions. The second pillar from the left in (f)–(g) corresponds to the tallest RB@Z7-NS pillar in (a)–(d). (h) FE-SEM of images in (f)–(g).

Our fourth strategy was to expand printing in 3-dimensions using RB@Z7-NS. Initial static printing and increased layering of small circles or rectangles revealed frequent collapse of structures (Figure S14). By dynamically controlling the focus ratio, however, it was possible to prevent collapse and increase material addition to achieve cylindrical micropillars (Figure 6). For pillar creation, a low focus ratio was used to maximize material deposition while reducing the risk of pillar collapse due to high gas flow. After an initial 1 min deposit both sheath flow rate and atomizer flow rate were reduced to enable increased pillar height. Pillars achieved varying heights, depending on the parameters used, with a maximum height of 430 μm (Figure 6a–h). For the tallest pillars, the following settings were used; initial focus ratio 0.675 (sheath flow rate 27 sccm, atomizer flow rate 40 sccm), secondary focus ratio 0.5 (sheath flow rate 10 sccm, atomizer flow rate 20 sccm).

The pillars emitted in the orange-red region (Figure 6g) are seen in the average emission spectra (Figure 6d) and cross-section plane image (Figure 6b). A 3D-stack analysis of the pillar revealed that fluorescence was concentrated at the top of the structure (along with the aggregated base particles) (Figure 6b). This may be a waveguide effect, generating intensity at the edges of the structure as observed in luminescent coumarin microfibers.^51^ Overspray is a common side-effect of pillar formation using AJP.^30,52^ While only a proof of concept was demonstrated in this study, further optimization of parameters, along with the use of supporting sacrificial templates (e.g., hydrophilic polymer molds), may increase the control of structure growth.

### Achieving White-Light Emission

**Figure 7:**
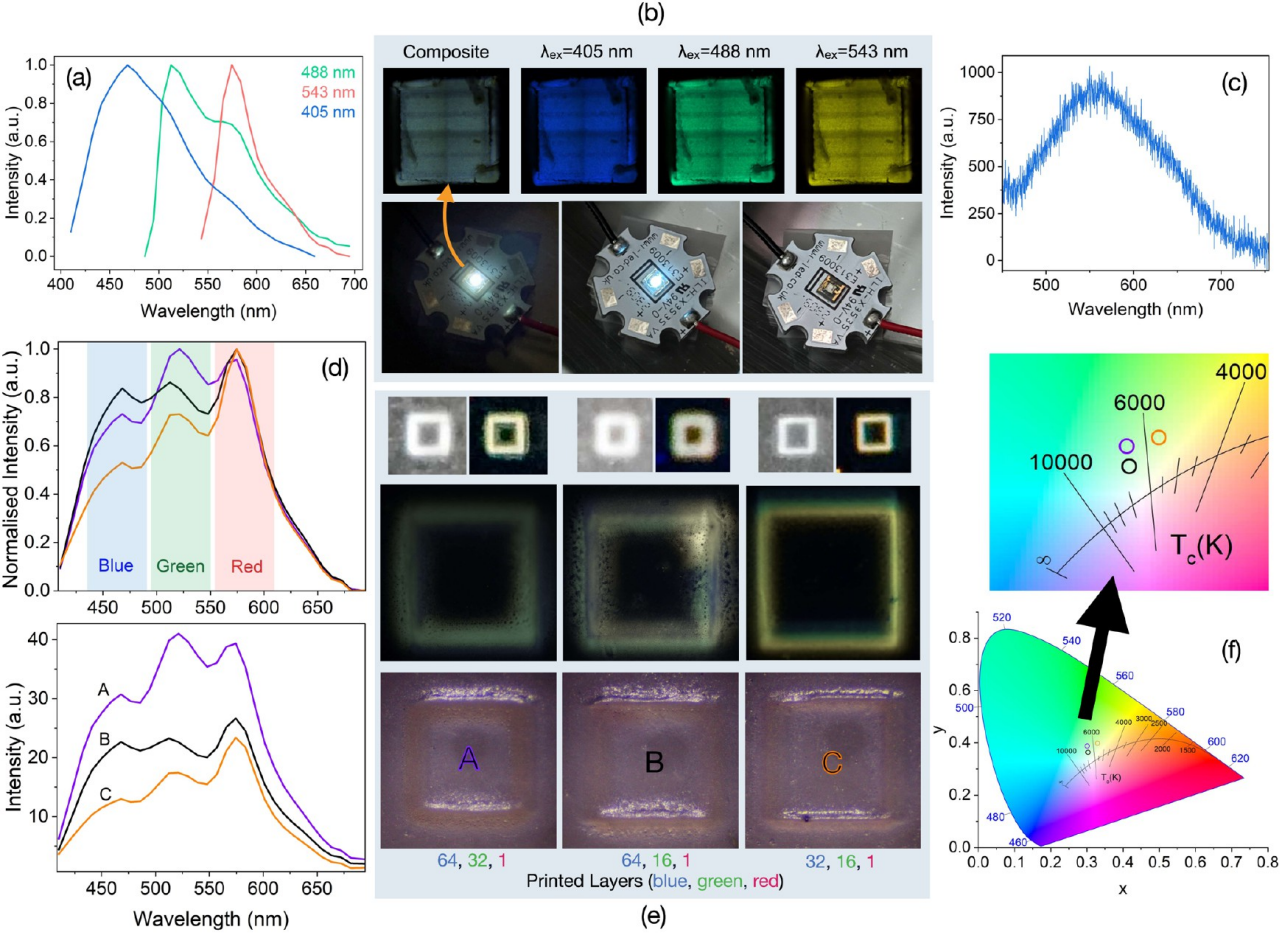
(a) Emission spectra of MC + F + RB@Z7-NS white-light-emitting ink on 365 nm emitting UV diode at various laser excitations (3 nm resolution). (b) Corresponding diode print (3.5 mm × 3.5 mm) microscopy images (top) with a combined average image (left). Images are a combination of four consecutive scans stitched together, causing the crosshair artifact. Bottom: Images of LMON-coated diode (3.4 V) off (right) and on (left). (c) Emission spectra of the diode when on. (d) Emission spectra of triple LMON layered WLE prints (3 nm resolution). (e) Images taken with a digital camera (top) and via microscopy (bottom) of corresponding prints (500 μm × 500 μm). (f) Chromatography of the layered prints.

Finally, two approaches were used to demonstrate white-light-emitting (WLE) LED (WLED) applicability of printing. For one, we coated a single thin layer of a white-light-emitting triple guest MC + RB + F@Z7-NS (previously reported) onto a flat square LED diode (3.5 mm × 3.5 mm, λ_em_ = 365 nm, 1 W at 3.4 V). Excitation with 405, 488, and 543 nm lasers confirms the presence of each guest emission band (MC at 460 nm, F at 515 nm, and RB at 580 nm) (Figure 7a,b). When operated at 3.4 V the LED glows white, most visible under darker conditions (Figure 7b). The emission spectra from the diode (Figure 7c) show a broadband of emission between 400 and 800 nm.

The synthesis of the triple guest WLE Z7-NS to achieve ideal white light is highly sensitive to guest concentration during synthesis; therefore, we also sought to develop a more robust WLE Z7-NS thin film methodology using additive manufacturing principles with our blue, green, and red MON emitter inks (MC, F, or RB@Z7-NS, respectively). Based on emission intensities from earlier strategy studies, a ratio of 64:32:1, 64:16:1, and 32:16:1 for blue/green/red Z7-NS emitter layers were selected. This produced prints of emission with CIE coordinates of (0.30, 0.38), (0.31, 0.36), and (0.32, 0.39) respectively (Figure 7d–f). Photographs of the prints under UV confirm their white appearance, and fluorescence microscopy confirms this emission color from lambda scanning, highlighting the change in the warmness of emission across samples (Figure 7e). The second print, achieving the closest to ideal white, exhibits three emission bands of near equivalent intensity, while the warmer third print shows a stronger RB emission contribution than MC (Figure 7d). As expected, the lighter white emission of the first sample has a stronger contribution by F.

## Conclusions

Coupling the advanced techniques of aerosol-jet printing with nanosheets of metal–organic frameworks (MONs) as inks enabled a new strategy for effectively printing luminescent films with precise geometries and patterns at the micron scale. The morphology of the MONs overcame MOF printing limitations, allowing for atomization, effective continuous printing, and well-formed patterns of the particle aggregates. Being digitally controlled, AJP was able to generate highly articulated images from logo patterns to intricate concentric squares. Functionalizing MONs with luminescent guests provided fluorescence emission-capable inks across a spectrum of colors. By variation of the layering and combination of inks, the emission chromaticity and intensity could be carefully tuned to a desirable output, including white-light emission. Meanwhile, printing with distinct inks enabled multiemission color prints at both the micron and centimeter scale. These prints are dynamically responsive to excitation wavelength, offering a platform for encoding environmentally sensitive information. Tuning the AJP parameters carefully further enabled the formation of 0.43 mm high-luminescence 3D pillars that exhibited a degree of directional emission.

These findings are critical for the advancement of luminescent MOF materials into devices from OLEDs to security tags and indicators. The potential of the established technique, however, goes far beyond. Altering the functionality of the MONs used as ink, such as enabling electroluminescence, sensing capabilities, or upconversion, could enable a wide gamut of luminescent-based devices for sensing (pressure, environments, temperature, etc.), optoelectronics, and energy storage. Beyond luminescence, conductive MONs could create carefully printed circuitry that could be immediately integrated into miniature devices. Collectively, the tunability of MONs coupled with the customizability of AJP offers a new platform for the patterning of integrated functional surfaces across scales from centimeters to microns.

## Data Availability

The data that support the findings of this study are available in the Supporting Information of this article. Any other data will be made available upon request.

## Supporting Information Available

Optical microscope images (Figure S1); SEM images (Figures S2 and S9); excitation spectra (Figure S3); emission intensity (Figures S4 and S5); PET substrate excitation (Figure S6); 64-layer F + RB@Z7-NS (Figure S7); solid-state powdered RB@Z7-NS absorption spectra (Figure S8); F@Z7-NS (32 layers) with RB@Z7-NS (Figure S10); RB@Z7-NS, F@Z7-NS, and MC@Z7-NS (Figures S11 and S12); large-scale RB@Z7-NS borders (Figure S13); and single position static printing of RB@Z7-NS (Figure S14) (PDF)
