# Supplementary material for: Fine-Scale Aerosol-Jet Printing of Luminescent Metal–Organic Framework Nanosheets
Source: ACS Appl Mater Interfaces. 2024 Oct 4;16(41):56304–15. doi: 10.1021/acsami.4c10713 (PMC11492290; doi:10.1021/acsami.4c10713)
Supplement: Supplementary file 1 — am4c10713_si_001.pdf [file am4c10713_si_001.pdf]

Supporting Information

Fine-scale Aerosol-Jet Printing of Luminescent Metal-Organic Framework Nanosheets

*Dylan A. Sherman,[a] Erik Landberg,[b] Anjana Ramesh Peringath,[b] Sohini Kar-Narayan,[b] Jin-Chong Tan[a]**

[a] Multifunctional Materials & Composites (MMC) Laboratory, Department of Engineering Science, University of Oxford, Parks Road, Oxford OX1 3PJ, United Kingdom.

[b] Department of Materials Science & Metallurgy, University of Cambridge, 27 Charles Babbage Road, Cambridge CB3 0FS, United Kingdom.

** Corresponding author's e-mail: jin-chong.tan@eng.ox.ac.uk*

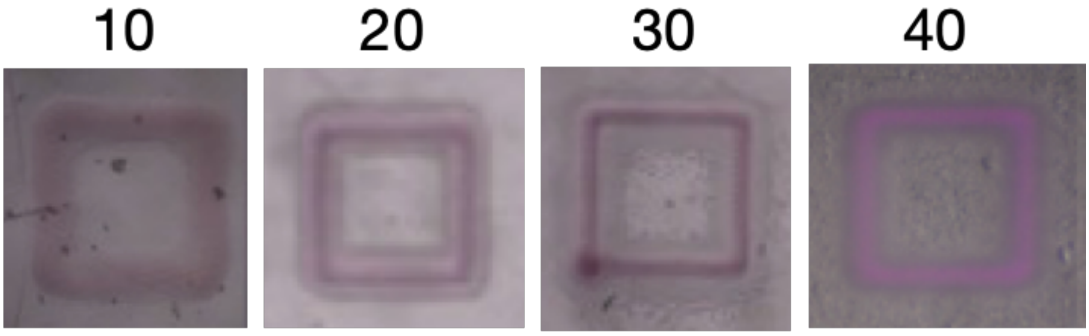

Figure S1. Optical microscope images of square perimeter prints (side length 500 μm) with RB@Z7-NS ink at various concentrations (10, 20, 30 and 40 mg/mL left to right).

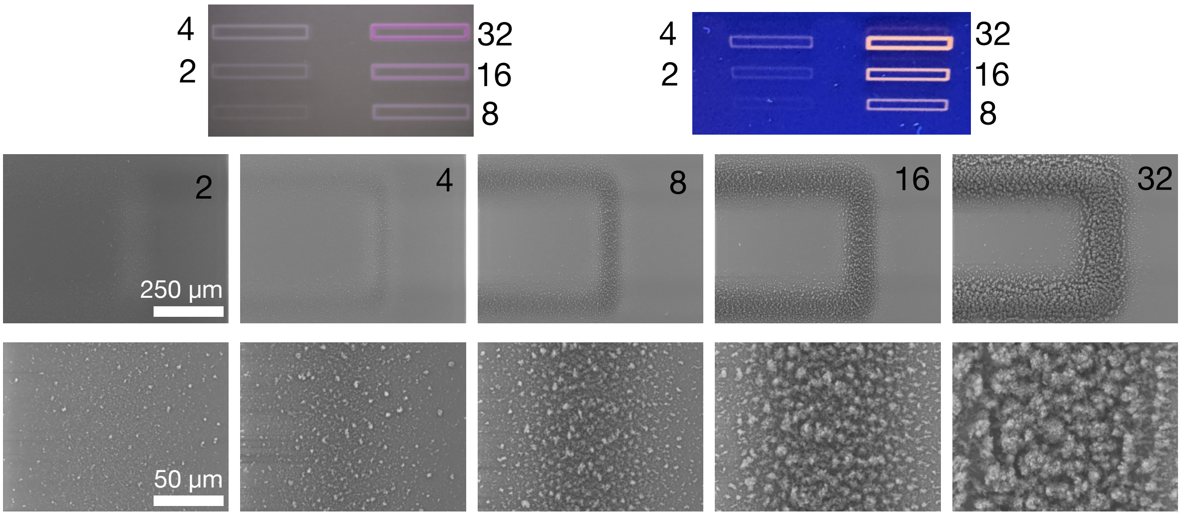

Figure S2. SEM of printed RB@Z7-NS(5 mm x 0.5 mm). Top: optical microscope images under ambient and UV (above).

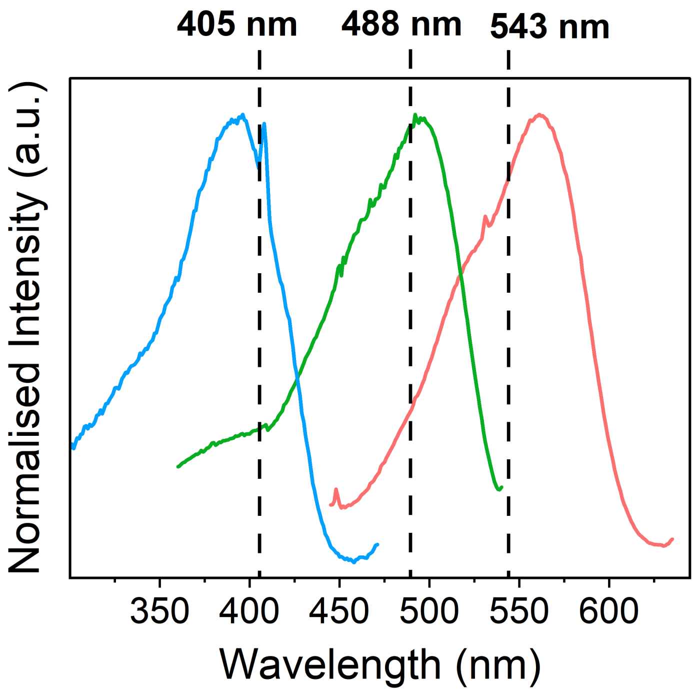

Figure S3. Excitation spectra of solid-state powder MC@Z7-NS (blue), F@Z7-NS (green), and RB@Z7-NS (red) overlayed with corresponding excitation laser wavelengths for fluorescence microscopy in this study.

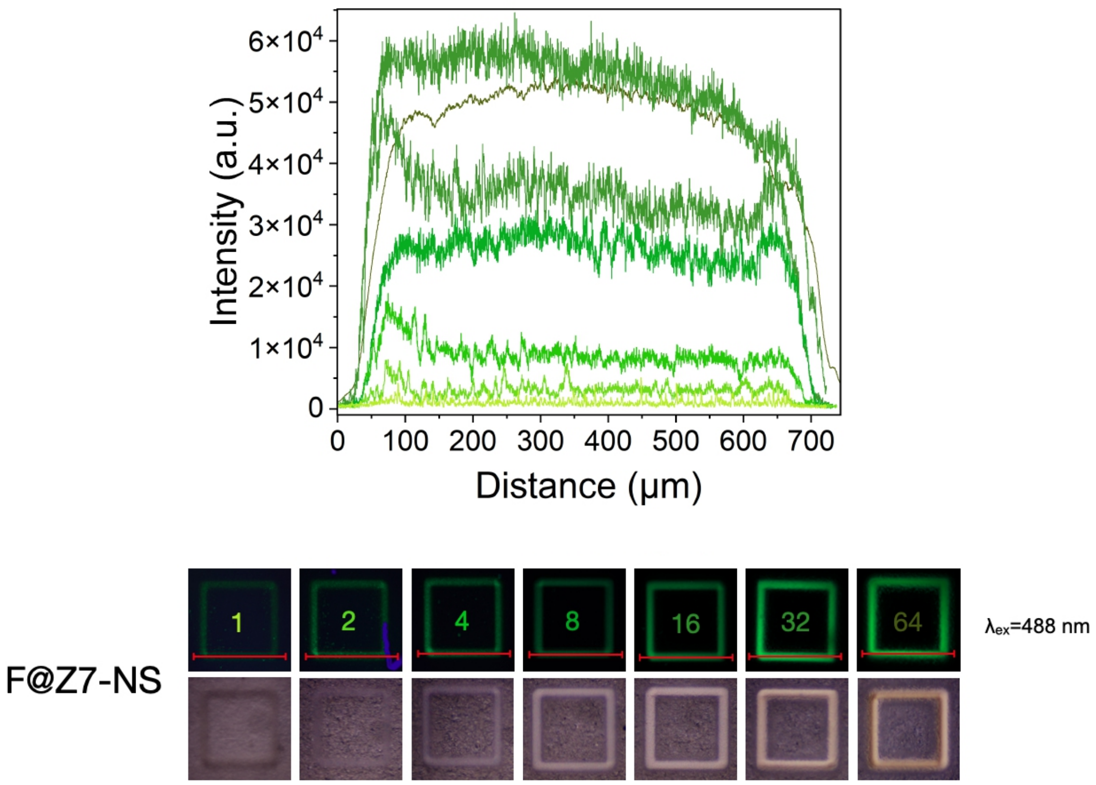

Figure S4. Emission intensity line scans across F@Z7-NS printed samples of varying layers. Line colours in graph correspond to colour of layer numbers indicated inside the prints (below) copied from Figure 1 for reference (each print 500x500 μm).

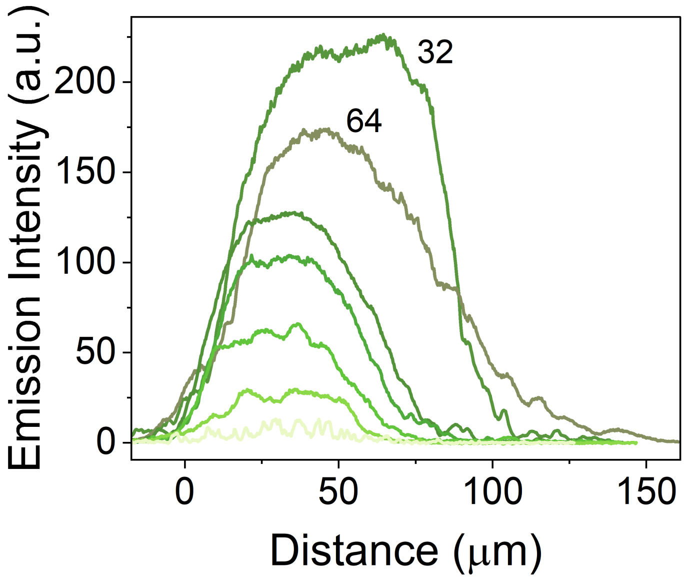

Figure S5. Emission intensity perpendicular to F@Z7-NS line prints, showing broad intensity across the line print, comparable to the line widths determined from profilometry.

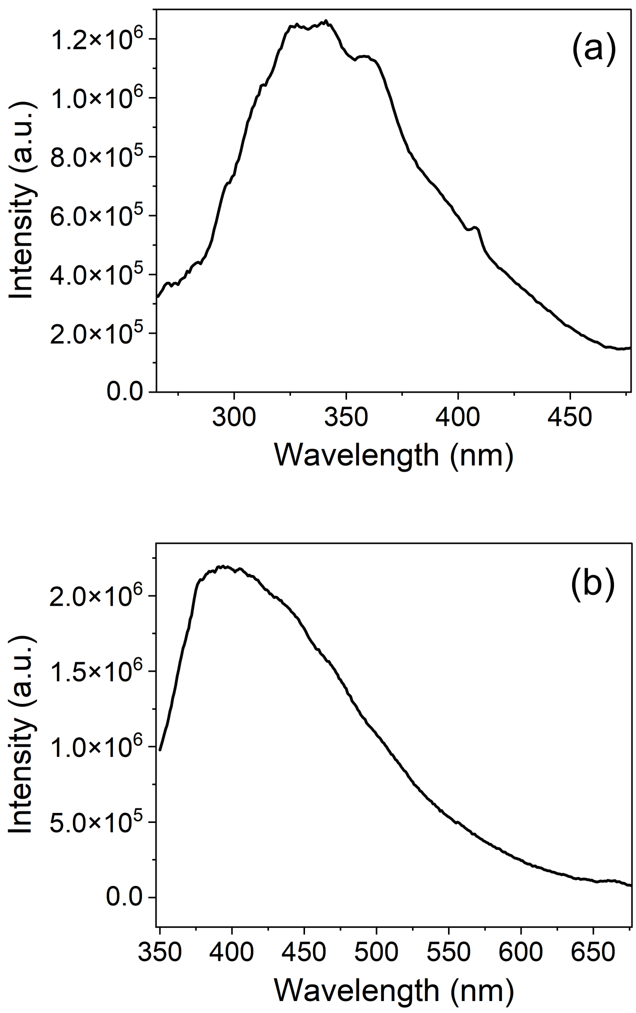

Figure S6. PET substrate excitation observed at 500 nm (a) and emission from excitation at 330 nm (b).

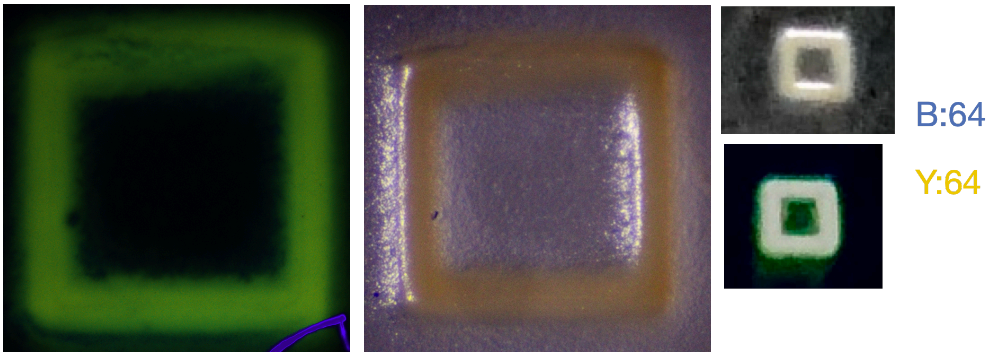

Figure S7. 64-layer F+RB@Z7-NS printed on 64-layer MC@Z7-NS (500x500 μm). Microscopy under UV (left) and ambient light (middle), digital photographs (right) under ambient (top) and UV (bottom) light.

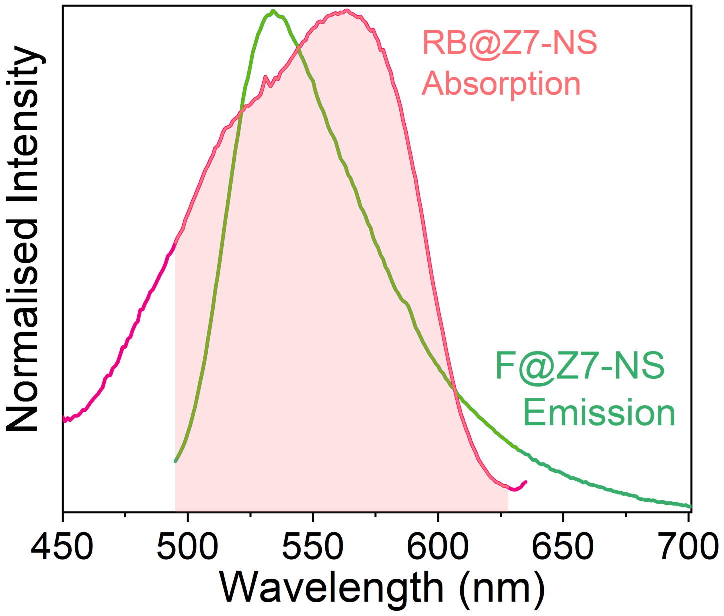

Figure S8. Solid-state powdered RB@Z7-NS absorption spectra overlayed with the emission spectra of F@Z7-NS, highlighting the overlapping of both bands.

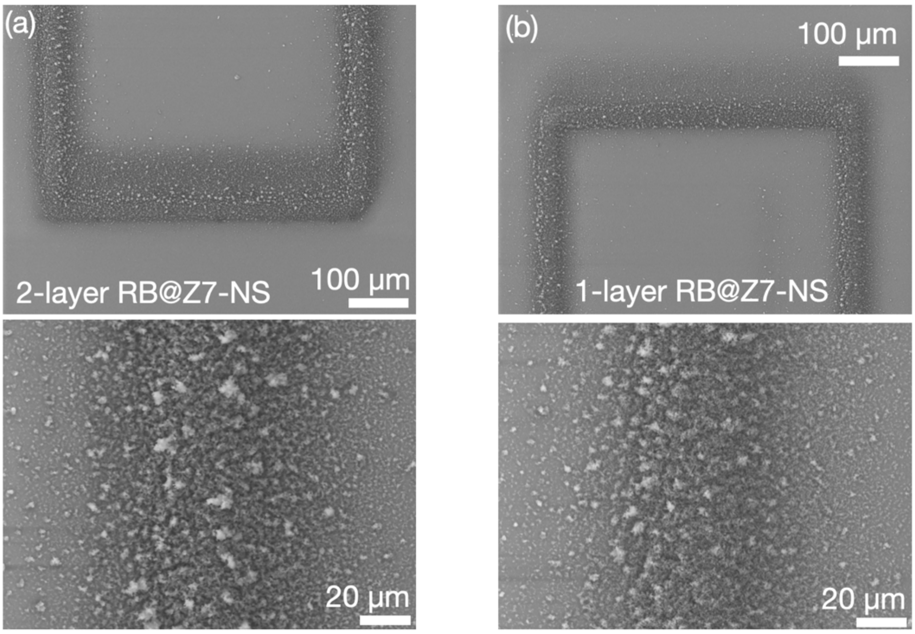

Figure S9. SEM of 2-layer RB@Z7-NS (a) and 1-layer RB@Z7-NS (b), each on 32-layer F@Z7-NS.

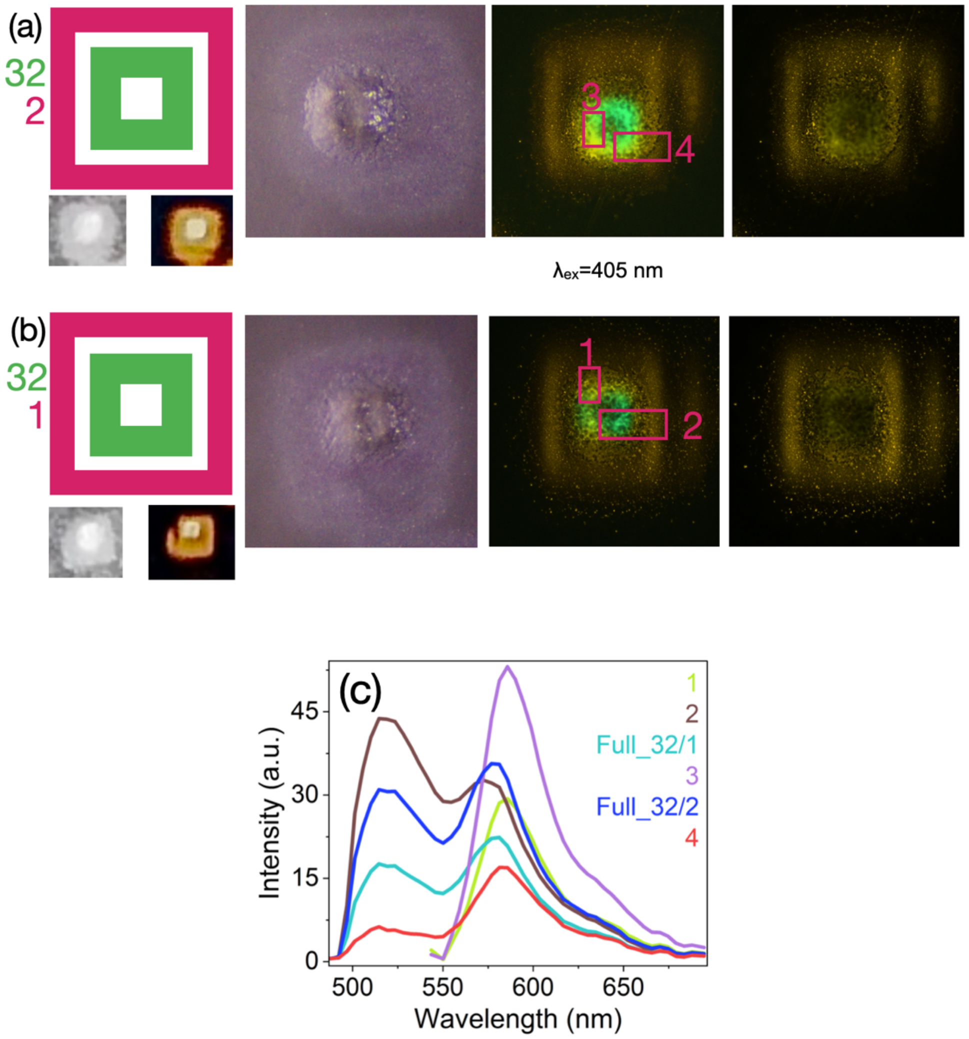

Figure S10. F@Z7-NS (32 layers) with RB@Z7-NS (2 layers for (a) and 1 layer for (b)) micron patterning comprised of concentric squares (300x300 µm for RB@Z7-NS and 120 x 120 µm for F@Z7-NS). Samples shown as diagrams (left, top), digital zoomed photographs under UV and ambient light (left, bottom), microscopy images under ambient (middle) and 405 nm laser excitation conditions (right). (c) emission spectra from fluorescence microscopy corresponding to highlighted squares in images.

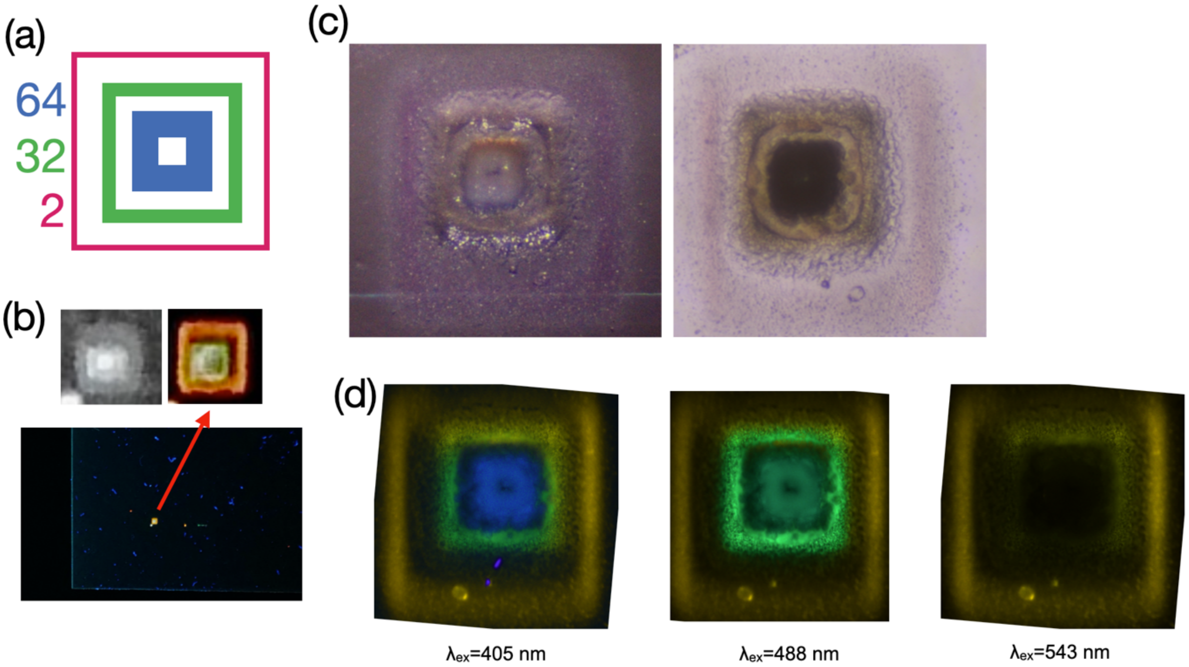

Figure S11. RB@Z7-NS (500x500 μm), F@Z7-NS (300x300 μm), and MC@Z7-NS (120x120 μm) multi-pattern printing comprised of concentric squares. Samples shown as diagram (a), digital zoomed photographs (b) under UV (right) and ambient light (left) including full image as seen by eye (bottom), microscopy images under ambient (c) and under various laser excitation conditions (d).

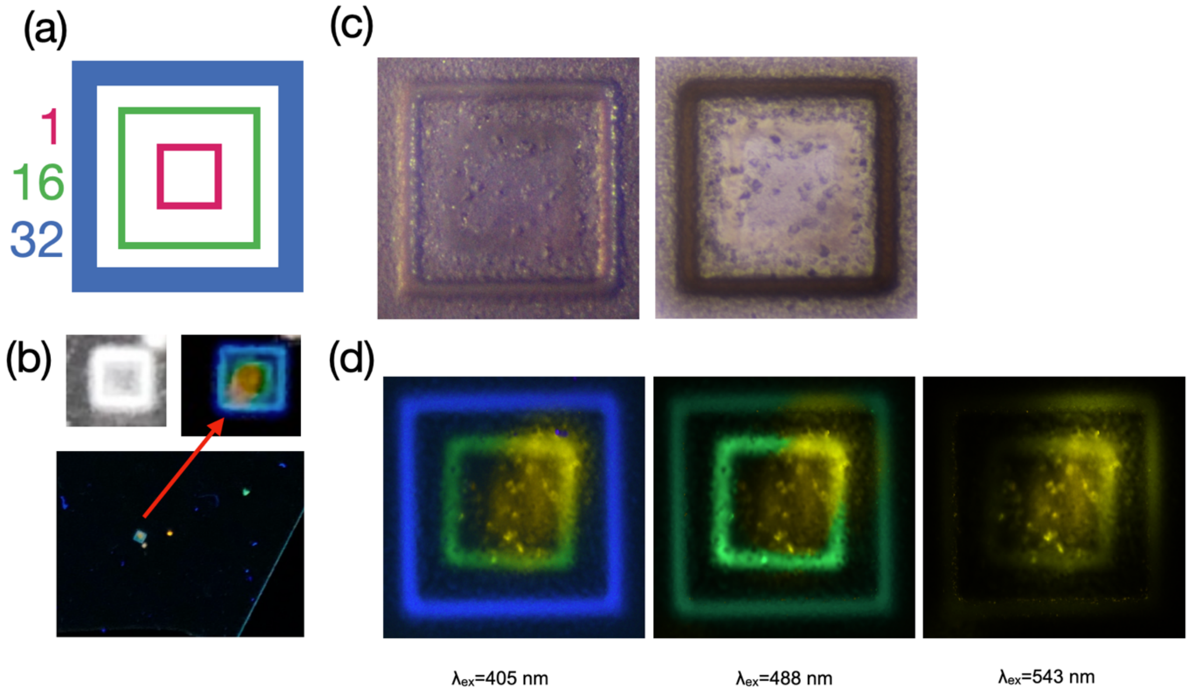

Figure S12. RB@Z7-NS (120x120 μm), F@Z7-NS (300x300 μm), and MC@Z7-NS (500x500 μm) multi-pattern printing comprised of concentric squares. Samples shown as diagram (a), digital zoomed photographs (b) under UV (right) and ambient light (left) including full image as seen by eye (bottom), microscopy images under ambient (c) and under various laser excitation conditions (d).

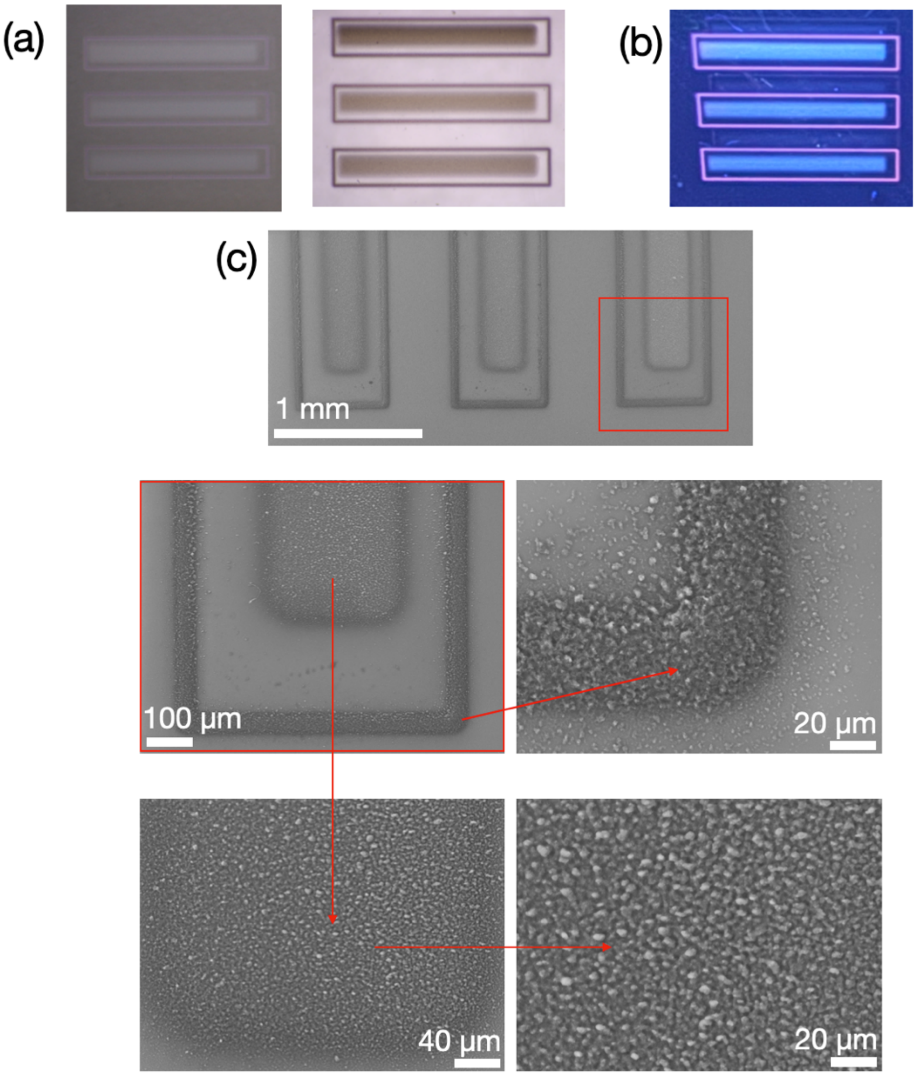

Figure S13. Large-scale RB@Z7-NS borders (5 mm x 0.5 mm) filled with F@Z7-NS. (a) microscope images under ambient conditions of prints. (b) prints under UV. (c) SEM of a single print edge, highlighting the border and central regions.

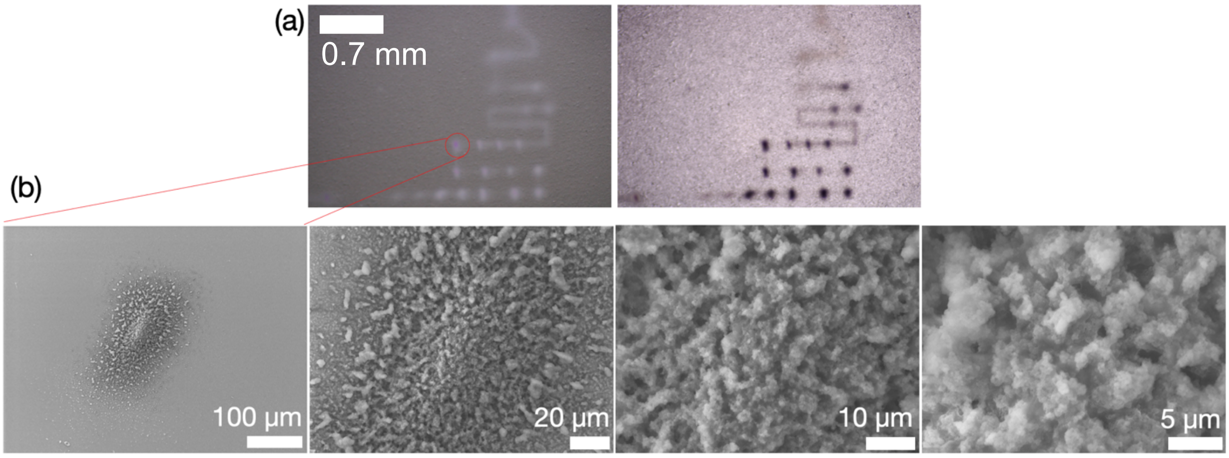

Figure S14. Attempts at single position static printing of RB@Z7-NS under optical microscope (a) and SEM (b).
